# Utility of C-2 (Cyclosporine) monitoring in postrenal transplant patients: A study in the Indian population

**DOI:** 10.4103/0971-4065.43691

**Published:** 2008-07

**Authors:** V. Thakur, R. Kumar, P. N. Gupta

**Affiliations:** Department of Laboratory Medicine, Batra Hospital and Medical Research Centre, New Delhi – 110 062, India; 1 Department of Nephrology, Batra Hospital and Medical Research Centre, New Delhi – 110 062, India

**Keywords:** Cyclosporine, graft rejection, immunosuppression, renal transplant

## Abstract

The study was planned and conducted to assess the benefit of C-2 levels (blood cyclosporine levels two hours postdosing) monitoring over trough (C0) levels (predosing) in postrenal transplant patients. The patient population included 34 postrenal transplant individuals (28 males and six females, mean age of 39.9 ± 12.3 years). The patients were first-transplant patients and were receiving a microemulsion form of cyclosporine A (CsA) as an immunosuppressant along with azathioprine and prednisolone. In addition, they were not on any enzyme inducer/inhibitor drugs, except for diltiazem. Timed collection of C0 and C-2 samples was done and the samples were immediately processed using the cedia cyclosporine plus assay kit. Estimation was done on a Beckman synchron CX5CE fully automated chemistry analyzer. Serum urea nitrogen and creatinine levels were checked. Poor graft survival was found in this population with 29.3% patients showing graft rejection. The graft rejection patients were assigned to two groups: group I with chronic graft rejection patients (17.6%) and group II with acute graft rejection patients (11.7%). Group III consisted of graft survival patients (70.7%). Mean ± SD was calculated for C0 and C2 levels. Individual values for C0 and C-2 were plotted on a scatter chart. C0 and C-2 levels were normalized by calculating them as the percentage of their targets (data not shown) and compared using the Kruskal Wallis one-way analysis of variance. C0 levels in all the three groups were within the recommended therapeutic range (150–300 ng/mL) (*P* < 0.182). Blood C-2 concentrations did not achieve the recommended target levels in these patients. One-way analysis of variance for C-2 values when expressed as the percentage of the target values did not show any significant difference between these groups (*P* < 0.84). No significant difference was found in C0 levels between groups I, II, and group III patients when expressed as the percentage of the target values (*P* < 0.182). The mean serum urea nitrogen level was 26.4 ± 8.1 mg/dL whereas the mean serum creatinine level was 1.79 ± 0.8 mg/dL. As is evident in the present study, the target C2 levels of cyclosporine dosage were not achieved whereas the C0 levels in all the three groups were within the optimum therapeutic range. As the incidence of graft dysfunction was also proportionately high, the importance of C2 monitoring is further highlighted on the basis of this study The contributory factors for levels lower than the target levels, such as the use of low doses of cyclosporine and interacting drugs should be carefully monitored in clinical practice. The achievement of optimum levels of C-2 may help in reducing the higher incidence of graft rejection in these patients. This practice is of equal importance in reducing cyclosporine-related renal toxicity, a rather irreversible process.

## Introduction

Cyclosporine is well established as a posttransplant immunosuppressant and has improved graft survival by about 15–20%. Drugs such as cyclosporine which have a low therapeutic index, require individualized monitoring of blood concentrations. Cyclosporine trough level monitoring is currently in use but interpatient variability in CsA exposure is greatest during the absorption phase (0–4 h) and adequate exposure during this phase is critical for its efficacy.^1^ Hence, C-2 monitoring is recommended in posttransplant patients in whom measurement of cyclosporine levels is done by taking a single sample two hours after dosing. The recommendation is based on CsA being referred to as a critical dosage drug with a narrow therapeutic index. The aim of C-2 monitoring in these patients is to achieve the optimal dose with minimal rejection and toxicity.

## Materials and Methods

The study was conducted on 34 consecutive postrenal transplant patients after obtaining their informed consent. The patients were on cyclosporine A (CsA), azathioprine, and prednisolone for immunosuppression. The patient population included 28 males and six females of mean age of 39.9 ± 12.3 years. All recipients were first-transplant patients. Blood cyclosporine levels were analyzed in two samples, one at 0 hours, *i.e*., before taking the morning dose of cyclosporine (C0) and another at 2 hours ± 15 minutes postdose (C-2). Serum urea nitrogen and serum creatinine levels were also determined. The postrenal transplant period was important in every individual because recommended C-2 target levels were dependent on this period. Determination of blood cyclosporine levels and other biochemical tests were done on a Beckman Synchron CX5CE fully automated chemistry analyzer.

Cyclosporine was analyzed by using the cedia cyclosporine plus assay (Microgenics Corp. USA). The cyclosporine assay is based on the bacterial enzyme, β-galactosidase, which has been genetically engineered into two inactive fragments. These fragments reassociate to form a fully active enzyme that cleaves a substrate and generates a color change that can be measured spectrophotometrically. Cyclosporine in the sample competes with the cyclosporine that has been conjugated to one inactive fragment of β-galactosidase for the antibody binding site. If cyclosporine is present in the sample, it binds to the mouse monoclonal anticyclosporine antibody, leaving the inactive enzyme fragments free to form active enzymes. If cyclosporine is not present in the sample, the antibody binds to cyclosporine conjugated to the inactive fragment, thus inhibiting the reassociation of β-galactosidase fragments, and no active enzyme is formed. The amount of active enzyme formed and the resultant absorbance change is directly proportional to the amount of the analyte (cyclosporine) present in the sample.^2^

Graft rejection was diagnosed by histopathological examination of biopsy taken from grafted kidneys. Out of 34 recipients, ten patients had graft rejection (*n* = 10, 29.3%). These patients were assigned to two groups: group I consisting of chronic graft rejection patients (17.6%) having a transplant period >12 months, and group II consisting of acute graft rejection patients (11.7%) with a transplant period <12 months. The patients with graft survival were assigned to group III (70.7%). Mean ± SD was calculated for C0 and C2 levels. Individual values for C0 and C-2 were plotted as scattergrams. C0 and C-2 levels were normalized by calculating them as the percentage of their targets (data not shown) and compared using Kruskal Wallis' one-way analysis of variance.

### Statistical analysis

Kruskal Wallis' one-way analysis of variance was applied to compare C0 and C-2 values calculated as percentage of the targets.

## Results

**Fig. 1:**
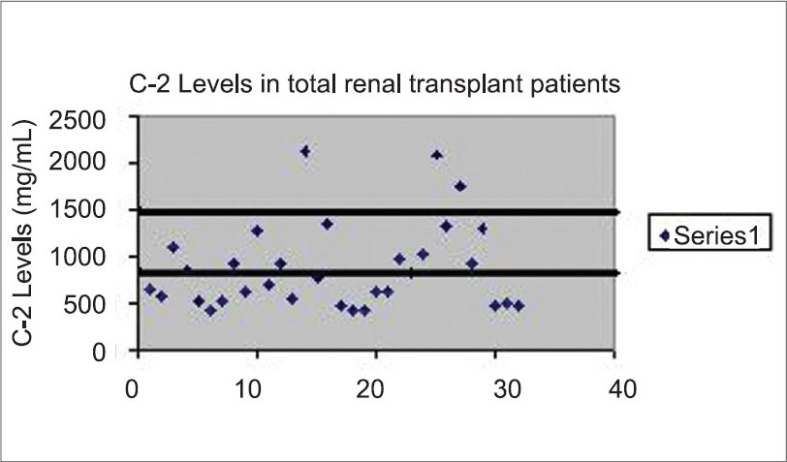
Distribution of individual C-2 values in the overall population of graft recipients. The target varies from 800 to 1500 ng/mL for these recipients depending upon their period of transplant.

It is very important to individualize the CsA dose schedule for each patient to optimize the pharmacological response. The overall mean C0 level in the patient population was 260 ± 96 ng/mL (target C0 levels = 150–300 ng/mL). The C-2 concentrations in these graft recipients did not achieve the optimal levels. The target values for C-2 were different for each patient as they varied according to their transplant period [Fig. 1]. The mean serum urea nitrogen level in these patients was 26.4 ± 8.1 mg/dL (normal range = 10–50 mg/dL) and the serum creatinine level was 1.79 ± 0.80 mg/dL (0.59–1.3 mg/dL).

**Fig. 2:**
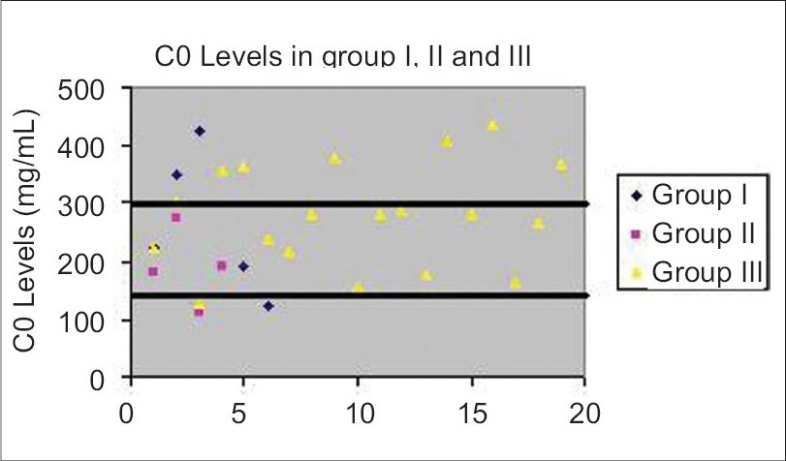
Distribution of individual C0 values in Group I, II and III recipients. The target range is 150–300 ng/mL.

**Fig. 3:**
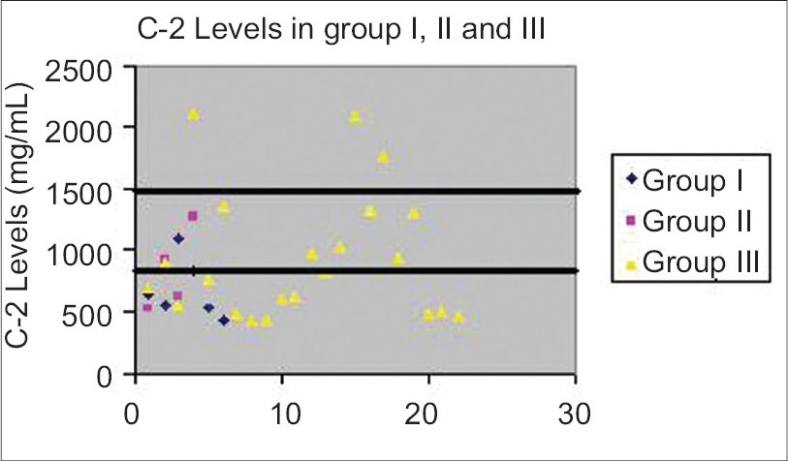
Scatter diagram depicting individual values for C-2 in group I, II and III patients. The target varies from 800 to 1500 ng/mL for these recipients depending upon their period of transplant.

The patients were grouped according to their graft rejection status. The graft rejection patients were assigned to group I with chronic graft rejection patients whereas group II consisted of acute graft rejection patients. Graft survival patients constituted group III. Individual values for C0 and C-2 in all the three categories were plotted as scatter diagrams [Figs. 2 and 3].

The mean level of C0 in group I patients was 262 ± 121.96 ng/mL, whereas the mean C0 concentrations were 188.50 ± 66.85 ng/mL and 279 ± 88.73 ng/mL respectively in groups II and III. The C0 levels in all the three groups were within optimal limits [Fig. 2]. For comparison with their targets, C0 levels were normalized by calculating the C0 values as percentage of the targets in all the three groups and compared using the Kruksal Wallis' test. No significant difference was found in any of the three groups (*P* < 0.182)

The mean C-2 concentrations in group I (683.83 ± 242.08 ng/mL) and II (836 ± 333.45 ng/mL) patients were lower than the mean C2 level of group III patients (938.31 ± 517.55 ng/mL). Distribution of individual C-2 values in all the three groups is depicted in Fig. 3, which shows that C-2 concentrations in patients of groups I and II did not achieve the target. Sixty percent of Group III patients also did not reach the optimal levels. The Kruskal Wallis' test for percentage of target C-2 levels did not show any significant difference in percentage C-2 values between all the three groups (*P* <0.84).

The serum urea nitrogen level in group I patients was 30.5 ± 7.08 mg/dL and the serum creatinine level was 2.58 ± 1.28 mg/dL. The mean serum urea nitrogen level in group II patients was 29.6 ± 12.8 mg/dL whereas the serum creatinine level was 2.0 ± 1.1 mg/dL.

## Discussion

The use of cyclosporine as a major immunosuppressant has revolutionized organ transplantation.^3^ The measurement of cyclosporine levels is an intrinsic part of the management of transplant patients because of inter- and intraindividual variations in its absorption and metabolism. The parent drug has a half-life of approximately eight hours and is metabolized by the cytochrome P450 liver microsomal enzyme systems. It is a common practice to measure the trough levels of cyclosporine (C0) rather than the peak (C-2) because of more consistent timing. However, extensive research on using C-2 values to monitor patients receiving cyclosporine has demonstrated reduced incidence and severity of rejection in *de novo* patients.^4^,^5^ Although many acknowledge the advantage of area-under-the-curve (AUC) monitoring, it has failed to gain widespread acceptance because of the practical difficulties in AUC measurement, both for the patient and the clinician. However, C2 levels best represent the AUC.

Cyclosporine is referred as a “critical dosage drug,” hence, it is very important to individualize the CsA dosages in every patient to optimize the pharmacological response of the drug. Cyclosporine has a narrow therapeutic index, which means that there is little difference in its levels that cause adverse effects and its levels that have therapeutic benefit, which makes it very important to achieve the target level. Measuring the blood concentrations aids in dosage individualization.^6^ Further arguments and recommendations focusing on C2 monitoring are reviewed elsewhere, including a consensus paper from the CONCERT group, as well as other reviews and recommendations. They have all supported the use of C2 levels as a more useful tool than C0 for CsA monitoring. The C2 level is where one gets the highest pharmacokinetic variability and hence, it is the one point that best characterizes absorption.^7^ Keeping in view the above facts, the present study was planned to determine the C0 and C-2 levels in all the recipients to adopt the C-2 monitoring for cyclosporine individualization in renal graft recipients. At present, the cyclosporine dosages were adjusted according to the trough levels, however, high graft rejection rates led us to think about adopting the alternate, precise, and accurate drug monitoring procedure of C-2 monitoring. The utility of C-2 monitoring was reviewed in contrast to the existing procedure (C0 monitoring) for cyclosporine dosage monitoring. Our patient population included 20% patients with early transplants (posttransplant period < 12 months) and 80% with late transplants (posttransplant period > 12 months).

The results of the study revealed that the C-2 concentrations in almost all patients did not achieve the recommended optimal target levels. The very high graft rejection rate in these patients could be attributed to low blood C-2 levels. Similar findings were observed in other studies also. A prospective single trial on the evaluation of C-2 monitoring and its clinical benefits in 30 *de novo* liver transplant patients has shown that if neoral dosage was adjusted according to the C-2 target levels, 80% of the patients had achieved the target level by day 3, whereas all of them could achieve the optimal levels by day 5. An overall incidence of rejection of 7% was documented in these patients.^8^ Studies have shown that C-2 is the point that correlates best with the AUC(0–4). C-2 monitoring in transplant recipients is feasible and safe and can also reduce the drug cost and improve kidney functions.^9^ Dominguez *et al.* have evaluated the benefits of monitoring cyclosporine dosages by C-2 estimation in stable renal transplant patients. According to them, among long-term renal transplant patients monitored by C0 or C-3 who were switched to C-2 monitoring, 61% of the patients had values above the target and 17% had values lower than the target levels. Six months after the monitoring by C-2, renal functions tests were found to be stabilized and there was a 10% reduction in the mean CsA dose.^9^ We recommend the adoption of C-2 monitoring in renal transplant patients to have the best performance of the graft, low rejection rates, and low nephrotoxicity. The reduction in drug cost is another benefit of the C-2 monitoring strategy. Our graft recipients and survivors deserve more than the precise monitoring of their life-saving drug.

## Conclusions

As is evident in the present study, target C2 levels of cyclosporine dosage were not achieved whereas the C0 levels in all the three groups were within the optimal therapeutic range. As the incidence of graft dysfunction was also proportionately high, the importance of C2 monitoring is further highlighted on the basis of this study. The contributory factors for a level lower than the target one, such as the use of low doses of cyclosporine and interacting drugs should be carefully monitored in clinical practice. The achievement of optimal levels of C-2 may help in reducing the higher incidence of graft rejection in these patients. This practice is of equal importance in reducing cyclosporine-related renal toxicity, a rather irreversible process.
